# An experimental study on the early diagnosis of traumatic brain injury in rabbits based on a noncontact and portable system

**DOI:** 10.7717/peerj.6717

**Published:** 2019-04-12

**Authors:** Jun Yang, Hui Zhao, Gen Li, Qisheng Ran, Jingbo Chen, Zelin Bai, Gui Jin, Jian Sun, Jia Xu, Mingxin Qin, Mingsheng Chen

**Affiliations:** 1 College of Biomedical Engineering, Army Medical University, Chongqing, China; 2 State Key Laboratory of Trauma, Burns and Combined Injury, Institute of Surgery Research, Third Military Medical University, Chongqing, China; 3 Department of Biomedical Engineering, Chongqing University of Technology, Chongqing, China; 4 Department of Radiology, Army Medical Center, Chongqing, China

**Keywords:** Closed cerebral hemorrhage, Traumatic brain injury, Magnetic induction phase shift, Noncontact, Prehospital diagnosis

## Abstract

Closed cerebral hemorrhage (CCH) is a common symptom in traumatic brain injury (TBI) patients who suffer intracranial hemorrhage with the dura mater remaining intact. The diagnosis of CCH patients prior to hospitalization and in the early stage of the disease can help patients get earlier treatments that improve outcomes. In this study, a noncontact, portable system for early TBI-induced CCH detection was constructed that measures the magnetic induction phase shift (MIPS), which is associated with the mean brain conductivity caused by the ratio between the liquid (blood/CSF and the intracranial tissues) change. To evaluate the performance of this system, a rabbit CCH model with two severity levels was established based on the horizontal biological impactor BIM-II, whose feasibility was verified by computed tomography images of three sections and three serial slices. There were two groups involved in the experiments (group 1 with 10 TBI rabbits were simulated by hammer hit with air pressure of 600 kPa by BIM-II and group 2 with 10 TBI rabbits were simulated with 650 kPa). The MIPS values of the two groups were obtained within 30 min before and after injury. In group 1, the MIPS values showed a constant downward trend with a minimum value of −11.17 ± 2.91° at the 30th min after 600 kPa impact by BIM-II. After the 650 kPa impact, the MIPS values in group 2 showed a constant downward trend until the 25th min, with a minimum value of −16.81 ± 2.10°. Unlike group 1, the MIPS values showed an upward trend after that point. Before the injury, the MIPS values in both group 1 and group 2 did not obviously change within the 30 min measurement. Using a support vector machine at the same time point after injury, the classification accuracy of the two types of severity was shown to be beyond 90%. Combined with CCH pathological mechanisms, this system can not only achieve the detection of early functional changes in CCH but can also distinguish different severities of CCH.

## Introduction

Traumatic brain injury (TBI) is a term indicating brain function changes or brain pathological changes caused by external forces acting on the head, which results in both high morbidity and mortality, particularly for people under 45 years of age (Zeiler et al., 2017; Namjoshi et al., 2013). A TBI occurs every 15 s, generating 1.7 million new brain injury victims per year in the US. These cases are annually responsible for 50,000 deaths and lead to 80,000 individuals with permanent disabilities (Prins et al., 2013). Motor vehicle accidents, falls and explosive blasts are the major causes of closed cerebral hemorrhage (CCH) (Wang et al., 2006; Pugh et al., 2016; Namjoshi et al., 2013).

As time progresses, CCH can be divided into two stages. One is the acute hemorrhage stage, with a rapid accumulation of blood, and the other is the chronic hemorrhage stage, with a gradual increase in intracranial pressure (ICP). Due to the dynamics of hematoma expansion, the primary damage occurs within minutes to hours after the injury and is a result of mechanical damage. Secondary injuries gradually occur as a consequence of ongoing cellular events that cause further damage. Many parallel pathological pathways exist, including: (1) Ion channel augmentation; (2) hypermetabolism; (3) excitotoxicity; (4) spreading depression; and (5) oxidative stress and inflammation (Kaur & Sharma, 2018; Davis, 2000; Namjoshi et al., 2013; Prins et al., 2013; Aronowski & Zhao, 2011; Chen, 2003). A delay in treatment will cause poor patient outcomes and death because of the accumulation of blood over time (Indraswari et al., 2012). Hence, there is a need for early techniques prior to hospitalization to diagnose occult injuries such as CCH from clinical observations.

The clinical golden standard for detecting CCH is computed tomography (CT), which can rapidly achieve both accuracy and quantitation in diagnosis. The main limitation of CT is that it is not suited for use in all regions, such as in remote villages, because of its bulk mass and operational complexity (Pandey et al., 2017; Amrhein et al., 2017). Although mobile CT units are developing, they are too rigorous for vehicles and rarely used roads, and their sensitivity and specificity are lower than those of a CT in a radiological chamber (Schwindling et al., 2016). Another common method of clinical management for CCH patients is ICP monitoring, which allows the real-time and accurate monitoring of ICP changes by implanting a microelectrode probe into the skull. There are high risks, however, for a secondary hemorrhage and infection (Giraudet et al., 2017; Liljacyron et al., 2018). Although CT imaging can achieve noncontact detection, it cannot perform real-time monitoring bedside due to its bulky size and low temporal resolution. ICP monitoring is the inverse of CT. Neither method can simultaneously achieve the requirements of noncontact and real-time monitoring to detect CCH. Currently, there is lack of safe and real-time diagnostic methods for CCH, or even TBI. In recent decades, many groups have reported detection methods used for the diagnosis of TBI, such as near-infrared spectroscopy (NIRS) and electrical impedance tomography (EIT). The detection principle of NIRS is to compare the near-infrared light absorption of the left and right brain hemispheres. The light value absorbed by the instrument is asymmetric on the two sides of the skull for TBI patients, which can demonstrate a hematoma on the side with the largest absorption. A detection sensitivity of 88% and a specificity of 90.7% for intracranial hemorrhage were shown by Robertson et al. (2010). However, the result is only for a hemorrhage larger than 3.5 ml, within 2.5 cm from the scalp. With no hemorrhage size limiting, the sensitivity decreases to 68.7% (Robertson et al., 2010). The NIRS has no advantage in the detection of the deep brain and small details. EIT is a functional imaging technique that images the electrical property distribution of the brain by safely injecting electrical currents into the surface of the skull and measuring the boundary voltages through specific electrodes. However, a shortcoming of EIT is that the cranium has a high electrical insulation that limits current penetration and restricts deep imaging of the brain without using invasive implanted electrodes (Koessler et al., 2017; Song et al., 2018; Manwaring et al., 2013).

Exploring the brain injury in a safe, noninvasive manner has become popular in recent decades. Microwave or radar technology can describe the development of TBI by monitoring the change of impendance characteristic (Mobashsher, Abbosh & Wang, 2014; Oziel, Korenstein & Rubinsky, 2017). Literally, monitoring the phase shift and the resonance frequency can attain more comprehensive information of dielectric properties which reflect the change of cranial contents (Gonzalez et al., 2013; Gonzalez & Rubinsky, 2006; Griffith et al., 2018; Kellner et al., 2018). In this study, a method based on the magnetic induction phase shift (MIPS) is proposed to detect high incidence of CCH in TBI for an early diagnosis. The MIPS technique is a safe, noncontact, real-time and new method for the detection of brain lesions that utilizes the changes in the dielectric properties of biological tissues to extract pathophysiology information of tissues. Since different types of tissues and organs exhibit different electrical properties, this method uses a single frequency signal that generates an alternating main magnetic field to pass through biological tissues that measures changes of tissue conductivity caused by the disturbing magnetic field (Jin et al., 2014a; Jin et al., 2014b). In our previous work, Jin et al. (2014b). designed a MIPS detection system based on a Tektronix signal source and a PXI platform to study intracranial hemorrhage in rabbits with a contralateral hemisphere cancellation coil by injection of a self-body blood model and discovered that the MIPS technique enables noncontact diagnosis and is sensitive to intracranial hemorrhage. But the model established by an intracranial injection of external blood differs from that of clinical TBI conditions. Li et al. (2017a) and Sun et al. (2016) showed that the MIPS technique is more sensitive than ICP monitoring when detecting brain lesions, demonstrating that the MIPS technique can achieve early detection in real-time. However, for TBI-induced CCH the ICP cannot increase immediately because of the effect of cerebral autoregulation (Cernak, 2006; Di Ieva, Schmitz & Cusimano, 2013). Therefore, this study aimed to investigate whether the MIPS method can perform an early diagnosis of TBI-induced CCH.

To obtain more information in the wide band in this study, we designed a magnetic induction brain monitor capable of measuring MIPS values from 300 kHz to 300 MHz. In addition, we established a cortical impact rabbit model based on the horizontal biological impactor BIM-II. Subsequently, with the self-designed monitor and coil sensor, we collected the MIPS data, which can reflect the change of mean brain conductivity caused by two CCH severity levels within 30 min of an injury. Additionally, the feasibility of this model was verified via CT images of three sections and serial slices 1 h after injury. Finally, the accuracy of CCH severity classification was determined based on a support vector machine (SVM) used to evaluate the performance of the MIPS method. We hope that the MIPS technique may serve as a new potential diagnostic method for prehospital diagnosis of CCH after a TBI, which can offer an early diagnosis program for CCH patients and carry out earlier treatments.

## Materials and Methods

### Detection system

**Figure 1:**
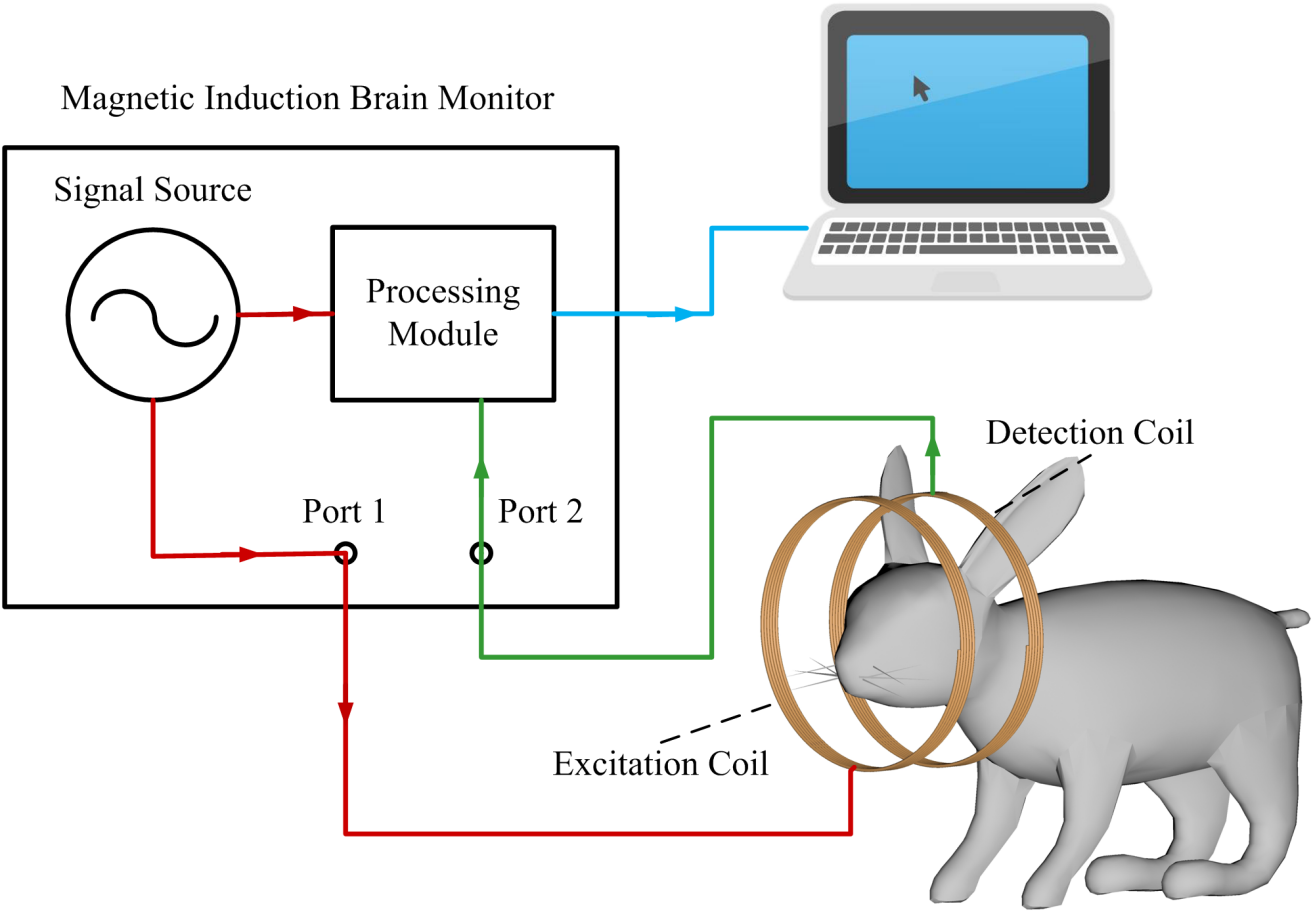
The schematic of the detection system.

The detection system of CCH primarily included a magnetic induction brain monitor (CNJY-2015; Tianda Instrument Company, Chengdu, China) and a coil sensor connected to two high frequency coaxial transmission lines. To better observe signal changes, we connected an external display screen, as shown in Fig. 1.

Magnetic induction brain monitor is an instrument with two ports whose excitation signal from source with a frequency range of 300 kHz–3 GHz and the direct digital frequency synthesis. The source formed two identical signals with the same amplitude, frequency range and initial phase via separation module, one of which received from Port 1 to Port 2 by the processing module, and another as reference signal output to it alike. Then, the processing module performed differential operations on above the two signals.

**Figure 2:**
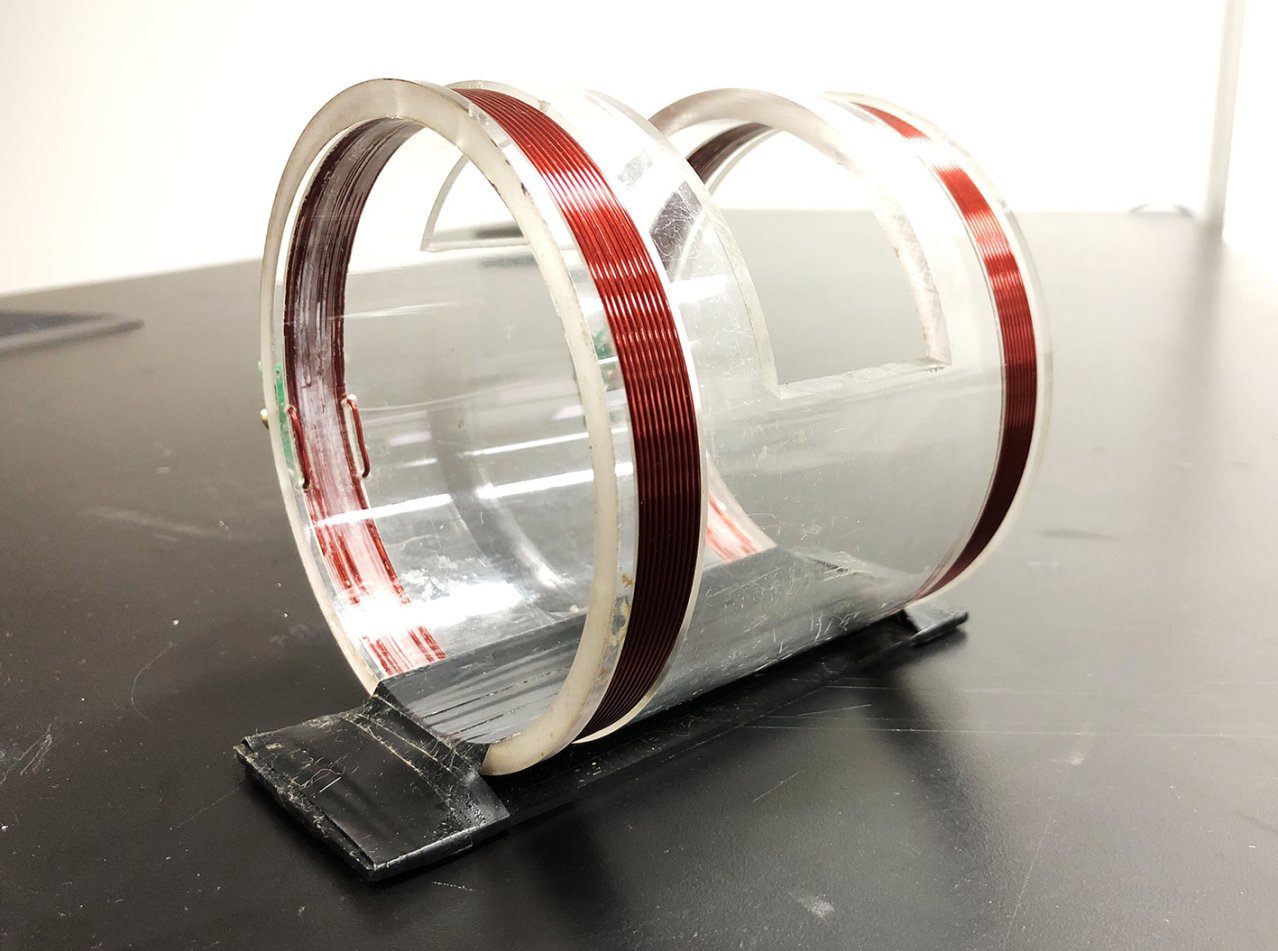
The two-coil sensor. Photograph by Jun Yang.

The coil sensor included the excitation coil and detection coil, as shown in Fig. 2. Both coils are wound with a one mm diameter AWG32 copper enameled wire at two terminals of the plexiglass tube with 10 winding turns. According to the size of a rabbit's skull, we designed the radius of the two coils to be 5.2 cm and the distance between the coils to be 10 cm, to fit the rabbits' heads into the coil.

### Detection principle

The excitation signal output from Port 1 generates a main magnetic field through the excitation coil through the output of Port 1. The main magnetic field can penetrate the target to generate an induced eddy current inside, thereby forming a disturbing magnetic field. The detection coil receives the vector addition of the main field and the disturbing magnetic field, named the superimposed magnetic field, which is transmitted to Port 2. The excitation signal partly transmitted from Port 1 to Port 2 via the target was defined as the transmission coefficient, and the remaining signal that did not pass through the target but was reflected to Port 1 was defined as the reflection coefficient that contained less information about the changes to the internal dielectric coefficient of the target than that of the transmission coefficient. Therefore, the data we used was taken from the transmission coefficient. Due to the directional coupler, the reflected signal and the transmitted signal do not interfere with each other.

$${\rm Reflection\; coefficient} = \displaystyle{{{V_{{\rm reflection}}}} \over {{V_{{\rm excitation}}}}} = r\ {\rm \angle\ \phi }$$

$${\rm Transmission\; coefficient} = \displaystyle{{{V_{{\rm transmission}}}} \over {{V_{{\rm excitation}}}}} = t\ {\rm \angle\  \psi }$$

where *r* and *t* are the amplitude of reflection coefficient and transmission coefficient, respectively. ϕ and ψ are the phase of reflection coefficient and transmission coefficient, respectively.

According to two-port network testing principle, the change in the impedance properties of the target will affect its conductivity, which is consistent with the MIPS theory (Griffiths, Stewart & Gough, 1999; Sun et al., 2014). In this study, the target is the brain, and there is a phase in the transmission coefficient called θ between the main magnetic field and the superimposed magnetic field (Nitsch, Rambousky & Tkachenko, 2015),

$${\rm \theta }\,{\rm = }\,{\rm arctg}\left( {P{{\rm \mu }_0}{\rm \omega \sigma }} \right)$$

and the changes in mean brain conductivity that are caused by an alteration in the proportion of intracranial tissue volume are a result of the shifts in phase (Δθ) of the current flow through the coil between *t*_1_ and *t*_2_.

$$\Delta {\rm \theta } = {\rm arctg}\left( {P{{\rm \mu }_0}{\rm \omega \Delta \sigma }} \right) = {{\rm \theta }_{{t_{\rm 2}}}} - {\rm \; }{{\rm \theta }_{{t_1}}}{\rm \; }$$

where *P* and μ_0_ are the target geometry and permeability of free space, both of which have a constant value, ω and σ are the angular frequency and conductivity, respectively, which both have variable values. Hence, Δθ is related to the frequency $f\left( {{\omega  \over {2\pi }}} \right)$ and the change in conductivity Δσ, when the circuit structure is stable.

Here, Δθ is the MIPS value that we use to reflect changes of the mean brain conductivity caused by CCH between *t*_1_ and *t*_2_.

$${\rm MIPS} = \Delta {\rm \theta } = {{\rm \theta }_{{t_2}}} - {\rm \; }{{\rm \theta }_{{t_1}}}{\rm \; }$$

### Measurement parameter

The frequency range of the excitation source was set from 300 kHz to 300 MHz. It was enough to set the number of scanning points to 1,001, to reduce the delay in data entry. The output power was adjusted to a maximum of 10 dBm for larger power transmission. The magnetic induction brain monitor can automatically trigger continuous scanning and sample data six times/min for 30 min. The data format that we saved to the system was set to amplitude and phase. After completing the above parameter settings, we performed the detection of rabbit CCH 30 min before and after injury.

### No-load measurement of detection system

**Figure 3:**
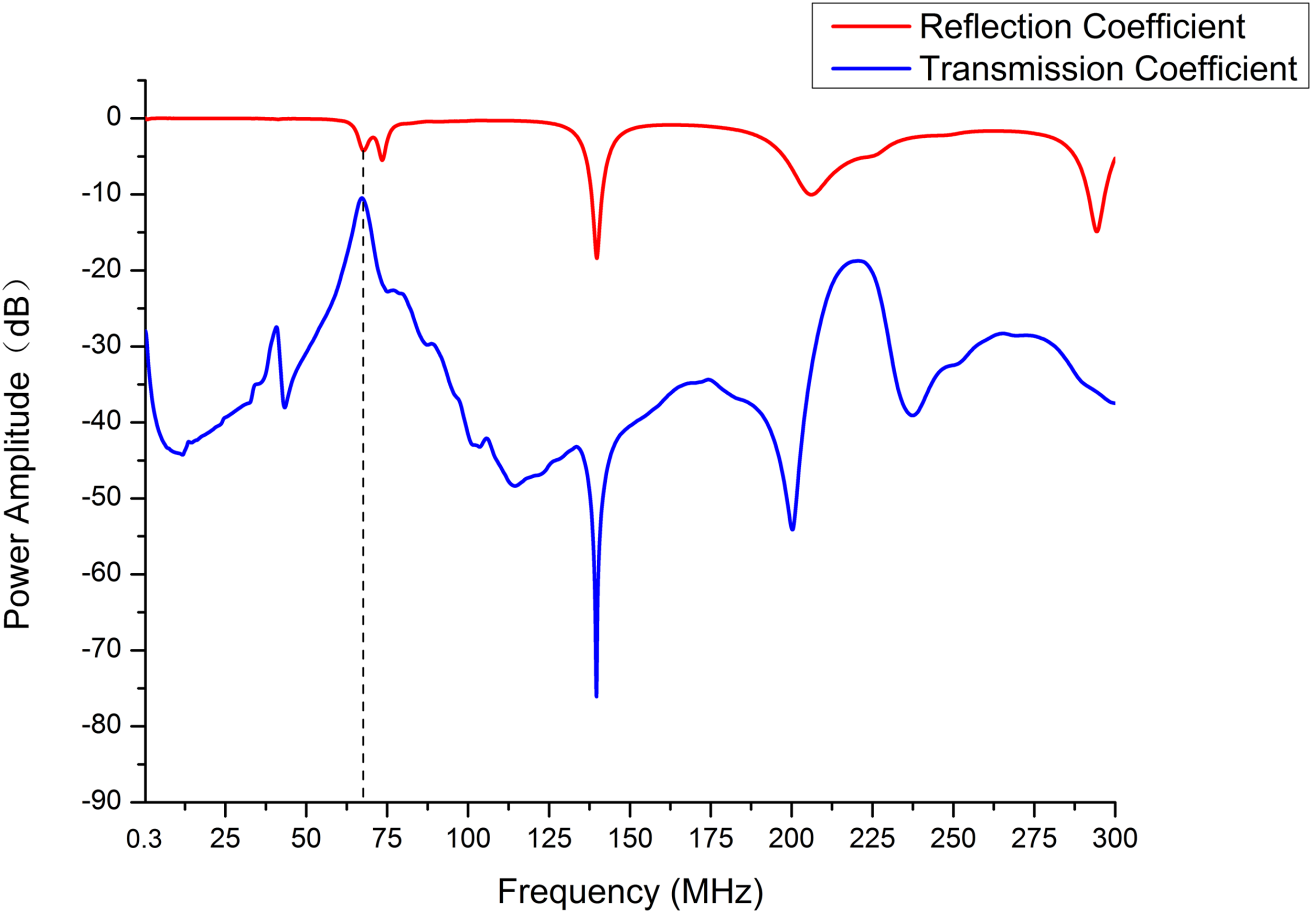
The power amplitude of reflection and transmission coefficient were measured in the frequency range of 300 kHz to 300 MHz. Dash line indicates maximum power amplitude of transmission coefficient and relatively lower power amplitude of reflection coefficient at 67.14 MHz.

Detection system maintained stable after short time of warming up under no-load circumstance (temperature drift less than 0.3°). Furthermore, in the frequency range of 300 kHz–300 MHz, we determined the amplitude-frequency characteristics of the reflection coefficient and the transmission coefficient, which was the power amplitude-frequency characteristics of the two ports when the coil sensor remained unloaded, as shown in Fig. 3. It showed that there was maximum power transmission, but lower power reflection, at 67.14 MHz, which illustrated that Port 2 received the maximum power transmission signal and a small reflection, that is, the signal source and the coil sensor formed the best impedance matching at this frequency. As a result, the MIPS value reached the highest sensitivity.

### Experimental design

All animal experiments were carried out in accordance with the guidelines of the Regulations on the Administration of Animal Experiment in Medical Research promulgated by the Ministry of Health of China. The protocol used was approved by the Laboratory Animal Welfare and Ethics Committee of the Third Military Medical University (SYXK-20170002). All efforts were made to minimize the pain of animals in the experiment. A total of 27 New Zealand white rabbits (2.2–2.7 kg, average body weight of 2.4 kg) were selected, and five died in the experiment. The remaining 22 rabbits were randomly divided into three groups (marked Nos. 1–20), composed of a group 1 (Nos. 1–10, *n* = 10), a group 2 (Nos. 11–20, *n* = 10) and a CT control group (Nos. 21–22, *n* = 2).

**Figure 4:**
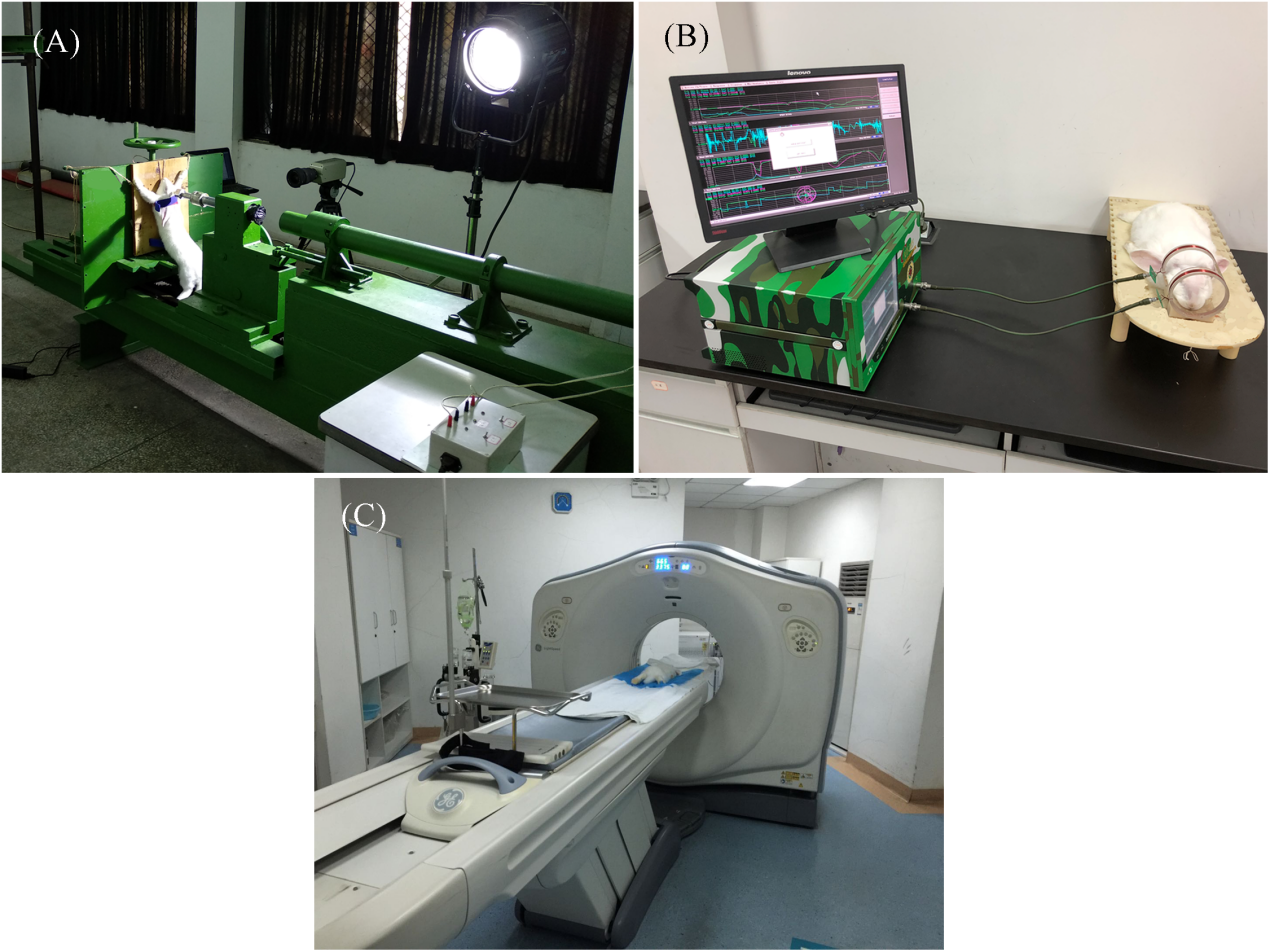
Experimental setup of CCH. Horizontal biological impactor BIM-II (A). CCH detection system in rabbits (B). CT image acquisition of a rabbit (C). Photographs by Jun Yang.

This experiment lead to rabbit CCH using the horizontal biological impactor BIM-II (Army Medical Center of PLA, Chongqing, China) and the cortical impact method. The impactor consisted of an air gun, a secondary hammer, a pedestal, a universal slab, a high-pressure gas source and a console with a maximum impact speed of 150 km/h. In group 1 and group 2, rabbits started with anesthetization via an ear vein injection of pentobarbital (3%, one ml/kg) and then the hair on the top of the head was removed. The rabbits under anesthesia were placed on the universal slab of the impactor. Then, by adjusting the place of the rabbit's head, the hammer was aimed at the point of the head with one mm in the back of the coronal line and six mm to the right of the sagittal line, as shown in Fig. 4A. Air pressure was set to 600 kPa for group 1 and group 2, respectively. The stroke time were recorded as 37 ms with a high speed camera (Phantom V4.3; Wayne, NJ, USA), respectively.

Figure 4B describes that each rabbit in group 1 and group 2 was placed into the coil sensor before and after the impact injury, positioned by point light locator with caliper tool to maintain consistent. According to the set parameters, the magnetic induction brain monitor continuously measured the MIPS value in the normal and injured conditions for 30 min with a data sampling rate of once every 10 s, controlled by system software. While measuring, the transient data of the MIPS value can be displayed on the monitor.

### CT analysis

Computed tomography imaging was used to get a semiquantitative consequence of the cerebral hemorrhage in rabbits before injury and 1 h after injury, as shown in Fig. 4C. A multislice spiral CT scanner (GE Lightspeed VCT-64 CT; Boston, Massachusetts, USA) was used to axially scan the head of prostrate rabbits, and the scanning parameters were set as follows: 140 kV, 500 mA, FOV 96.0 mm, rotation time 2 s/r, and a slice thickness of 0.625 mm. The sagittal plane and transverse plane of the rabbit head were reconstructed by the obtained CT data in an image postprocessing workstation (AW4.2; GE Medical Systems, Chicago, IL, USA).

### Statistical analysis

All the data were expressed as the mean ± standard deviation from 20 independent experiments in group 1 and group 2. At the selected frequency, the differences in the MIPS values from the scans before and after injury were evaluated using a paired *t*-test to distinguish whether there is a significant difference in the average deviation. Within 30 min, time correlation with the MIPS value was performed by the Pearson correlation analysis. At the same moment, the significant difference analysis of the MIPS values between group 1 and group 2 was performed using a paired Wilcoxon signed rank test. Statistical analysis was performed using SPSS version 19.0 (SPSS Inc.; Chicago, IL, USA), and *p*-values under 0.05 were considered significant.

## Results

### TBI model validation using CT images

**Figure 5:**
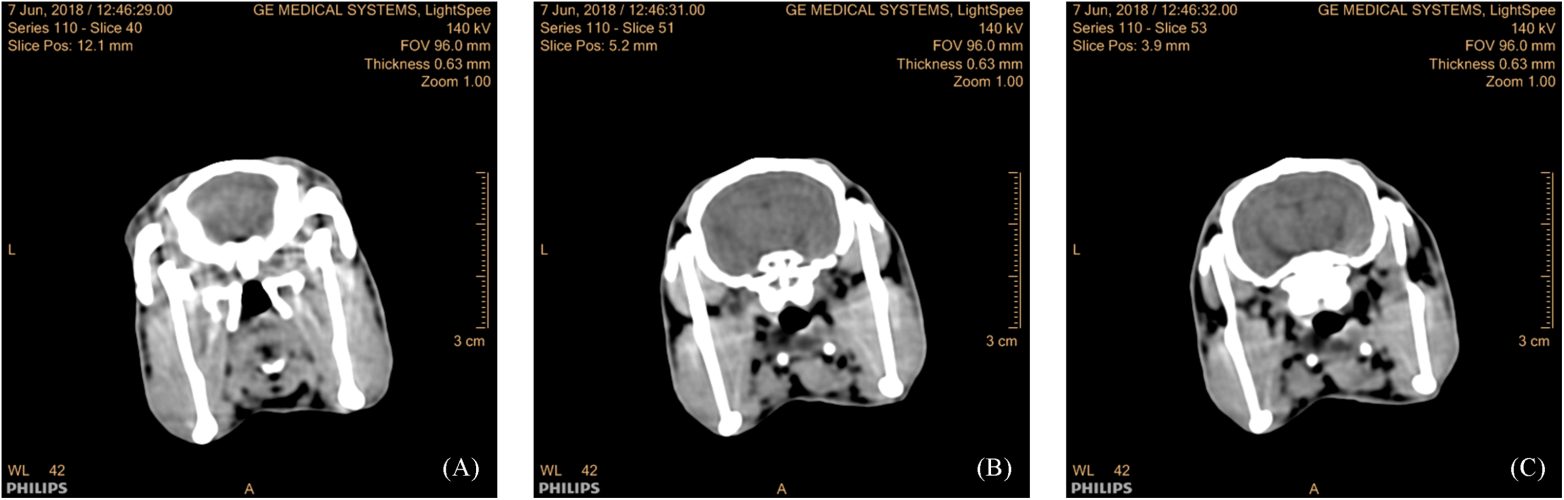
Three-layer coronal CT images of rabbits in the control group. Coronal images of the 40th slice (A), 51st slice (B), 53rd slice (C).

**Figure 6:**
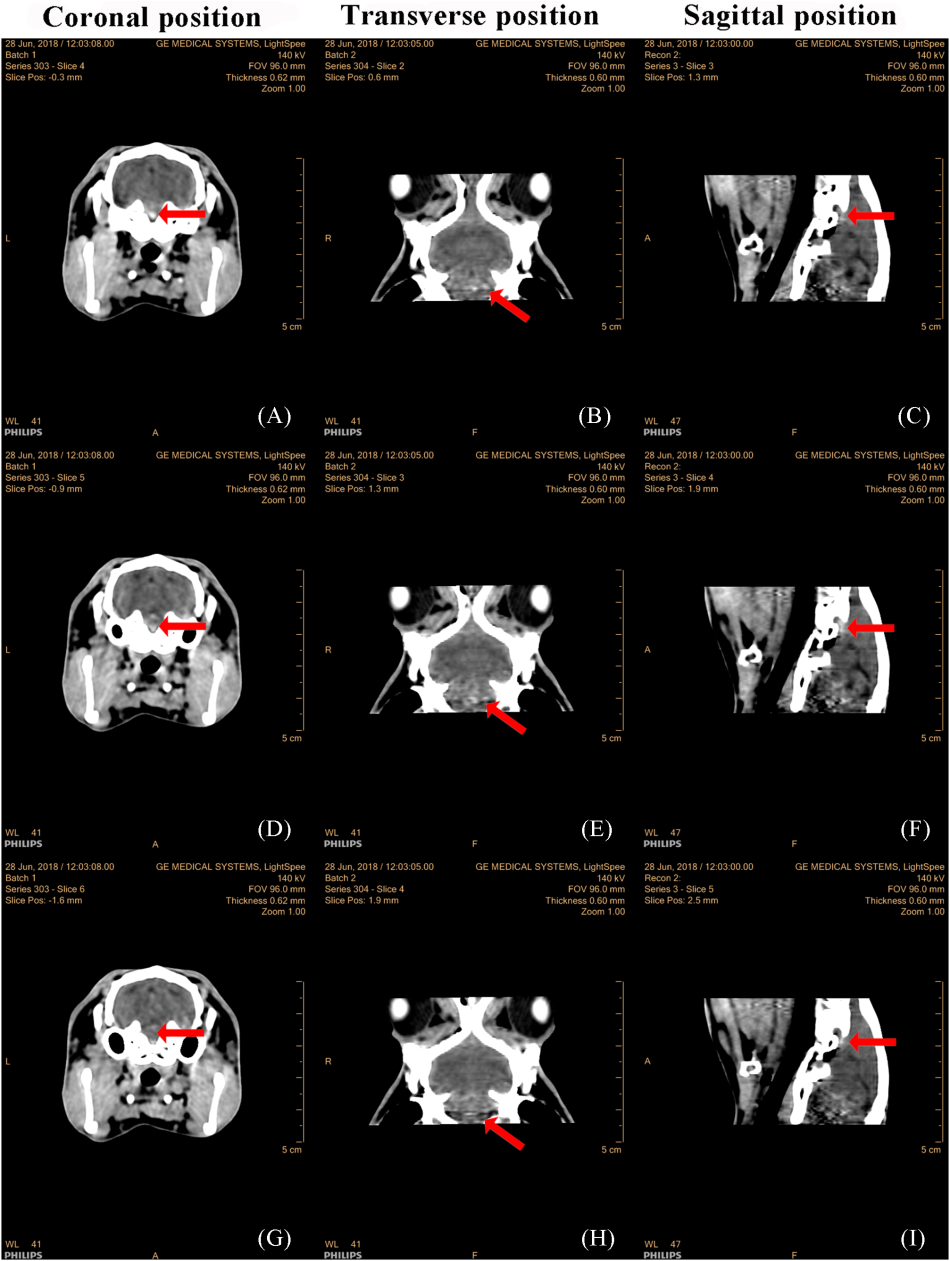
CT images of three serial layers and three sections in group 1, 1 h after injury by a 600 kPa impact. Coronal images of the 4th to 6th slice (A, D, G, respectively). Transverse images of the 2nd to 4th slice (B, E, H, respectively). Sagittal images of the 3rd to 5th slice (C, F, I, respectively).

**Figure 7:**
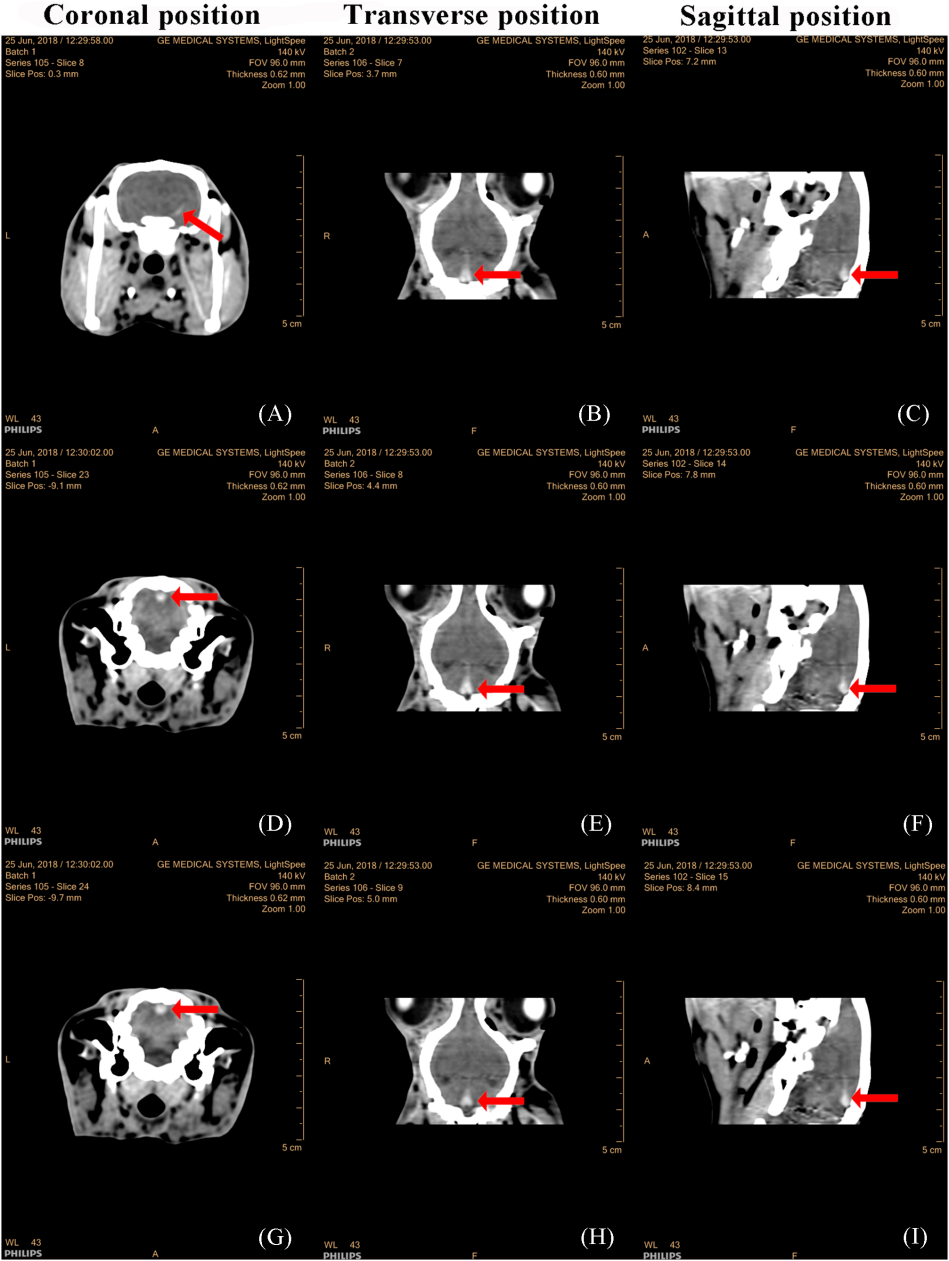
CT images of three serial layers and three sections in group 2, 1 h after injury by a 650 kPa impact. Coronal images of the 8th slice (A), 23th slice (B) and 24th slice (C). Transverse images of the 7th to 9th slice (B, E, H, respectively). Sagittal images of the 13th to 15th slice (C, F, I, respectively).

Figure 5 presented that there were no clear abnormalities in the brain of the three serial slices in the coronal images of the control group. In group 1 after 1 h of injury via a 600 kPa impact, there were obvious elliptical hemorrhages at the base of the skull in the images from three serial slices and three sections, as shown in Fig. 6. In group 2, after 1 h of injury via a 650 kPa impact, there were obvious strip hemorrhages in the left hemisphere and elliptical hemorrhages in the parietal lobe of serial three-slice and three sections of the images, as shown in Fig. 7. In the coronal position, the number of images with a hemorrhage was five in group 1, and 17 in group 2, revealing that CCH in group 2 had more and wider hemorrhages, based on the morphology and thickness of the hemorrhages. In the CT experiments, the feasibility of the CCH model, which can form a hemorrhage in the impact or hedging location and can lead to two different severity levels, was verified.

### MIPS detection result

In the study, five rabbits died after the impact injury, and only two healthy rabbits' CT images were excluded. The remaining 20 rabbits, which maintained a normal heart rate and breathing rate during the measurement period, contributed to the MIPS data. In measurements, the peak value of MIPS at all frequency points ranging from 300 kHz to 300 MHz was selected to be the source data for diagnosis, and the frequency point corresponding to the peak value was called characteristic frequency.

### MIPS in group 1

**Figure 8:**
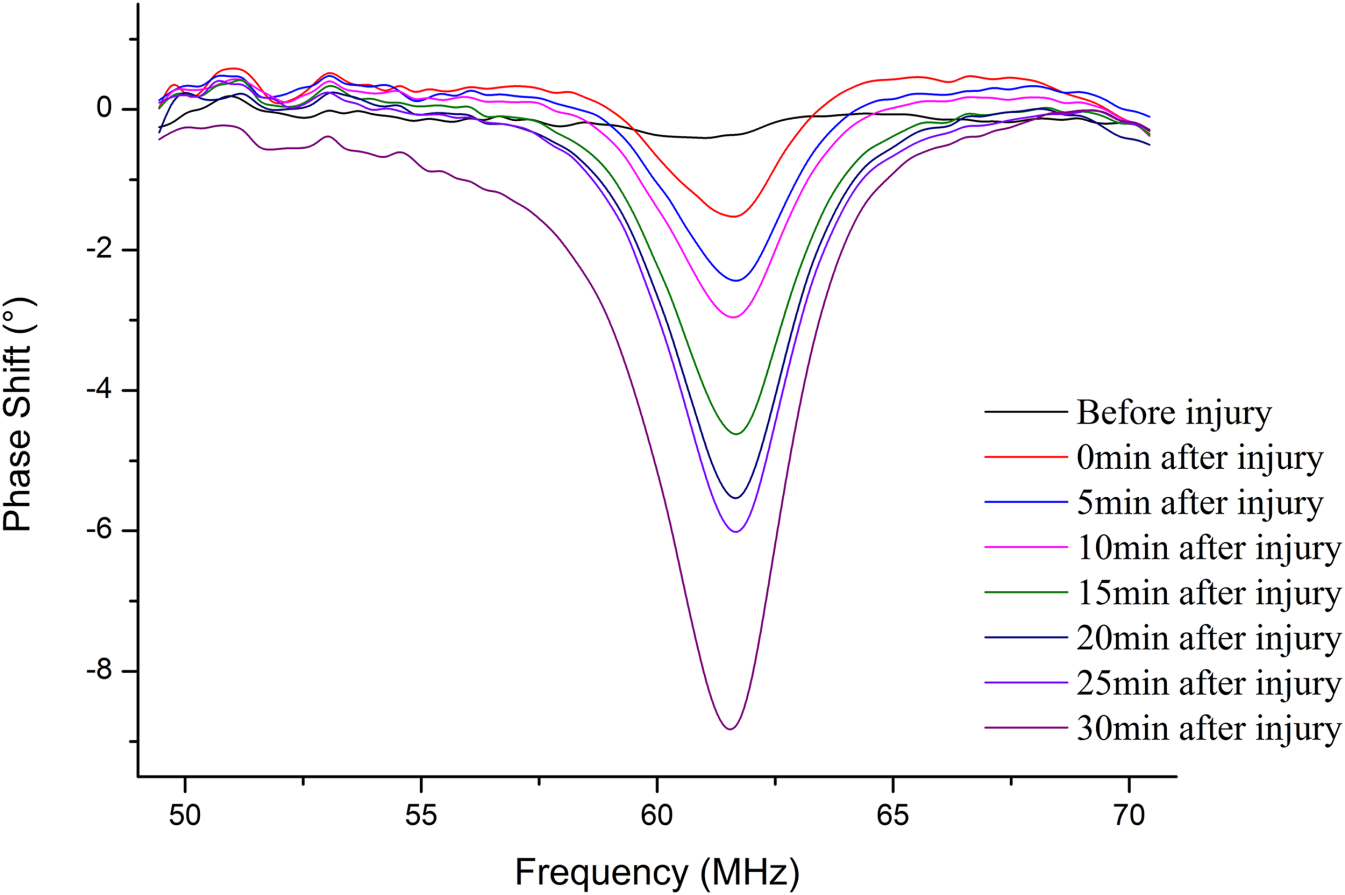
The MIPS curves were plotted before injury and 0 min, 5 min, 10 min, 15 min, 20 min, 25 min, 30 min after injury in the frequency of 50 MHz to 70 MHz.

Figure 8 shows that the MIPS value at characteristic frequency from No. 3 was related to time after injury in the 30 min period. More importantly, the MIPS value stably stayed at approximately 0° before injury, while the MIPS value decreased with time after injury.

**Figure 9:**
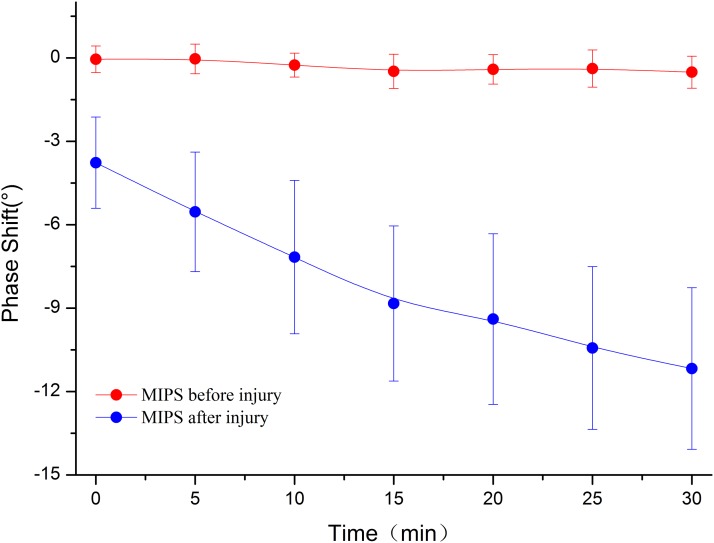
In group 1, the mean ± standard deviation MIPS curve of rabbits were declining within 30 min after injury compared to which before injury.

Figure 9 shows the mean ± standard deviation trends of MIPS values at 62.61 ± 1.32 MHz within 30 min before injury and 30 min after injury in group 1. The mean MIPS value was −3.77 ± 1.64° at 0 min and −11.17 ± 2.91° after injury and displayed a persistent downward trend. In contrast, there was no clear change to the mean MIPS value, which stayed at approximately 0° before injury (mean MIPS: −0.51 ± 0.58°). A paired *t*-test analysis with *p*-value < 0.05 for the same time of the mean MIPS value between before and after injury scans indicated that there was a significant difference between MIPS values before injury and after injury. This result demonstrated that MIPS values can discriminate CCH in group 1. The Pearson correlation coefficient between the mean MIPS value and time was −0.877 before injury (|r| < 0.9). In contrast, the Pearson correlation coefficient was −0.983 after injury (|r| > 0.9). This shows that the trend of the mean MIPS value is not well correlated with time before injury but is closely related to time after injury, which reflects that there was a continuous change in the brain during the measurement. The result indicates that the trend in MIPS values was consistent with the theory that as the pathological process progresses the hemorrhage increased, resulting in a decrease in the mean brain conductivity (Jin et al., 2014a; Laufer, Solomon & Rubinsky, 2012).

### MIPS detection results in group 2

**Figure 10:**
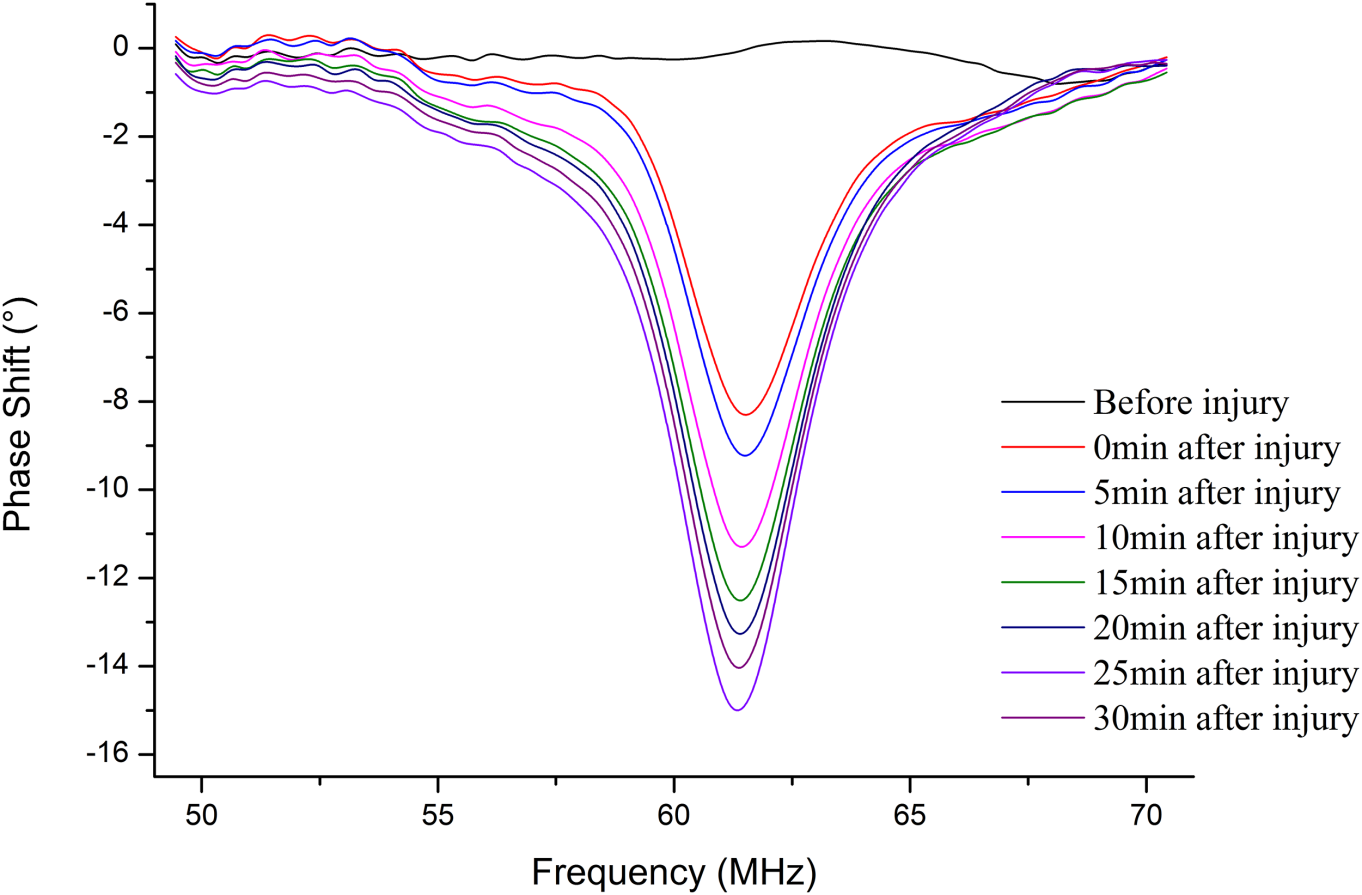
The MIPS curves were plotted before injury and 0 min, 5 min, 10 min, 15 min, 20 min, 25 min, 30 min after injury in the frequency of 50 MHz to 70 MHz.

Figure 10 shows that the MIPS value at characteristic frequency from No. 14 was related to time after injury in the 30 min period. Similar to group 1, the MIPS value was stable approximately 0° before injury. Unlike group 1, the minimum MIPS value appeared at 25 min, then increased until the end of the measurement. This indicates that the change in mean brain conductivity in No. 14 was not consistent from 0 to 30 min after injury, and there was an inflection point in the later stages of the measurement.

**Figure 11:**
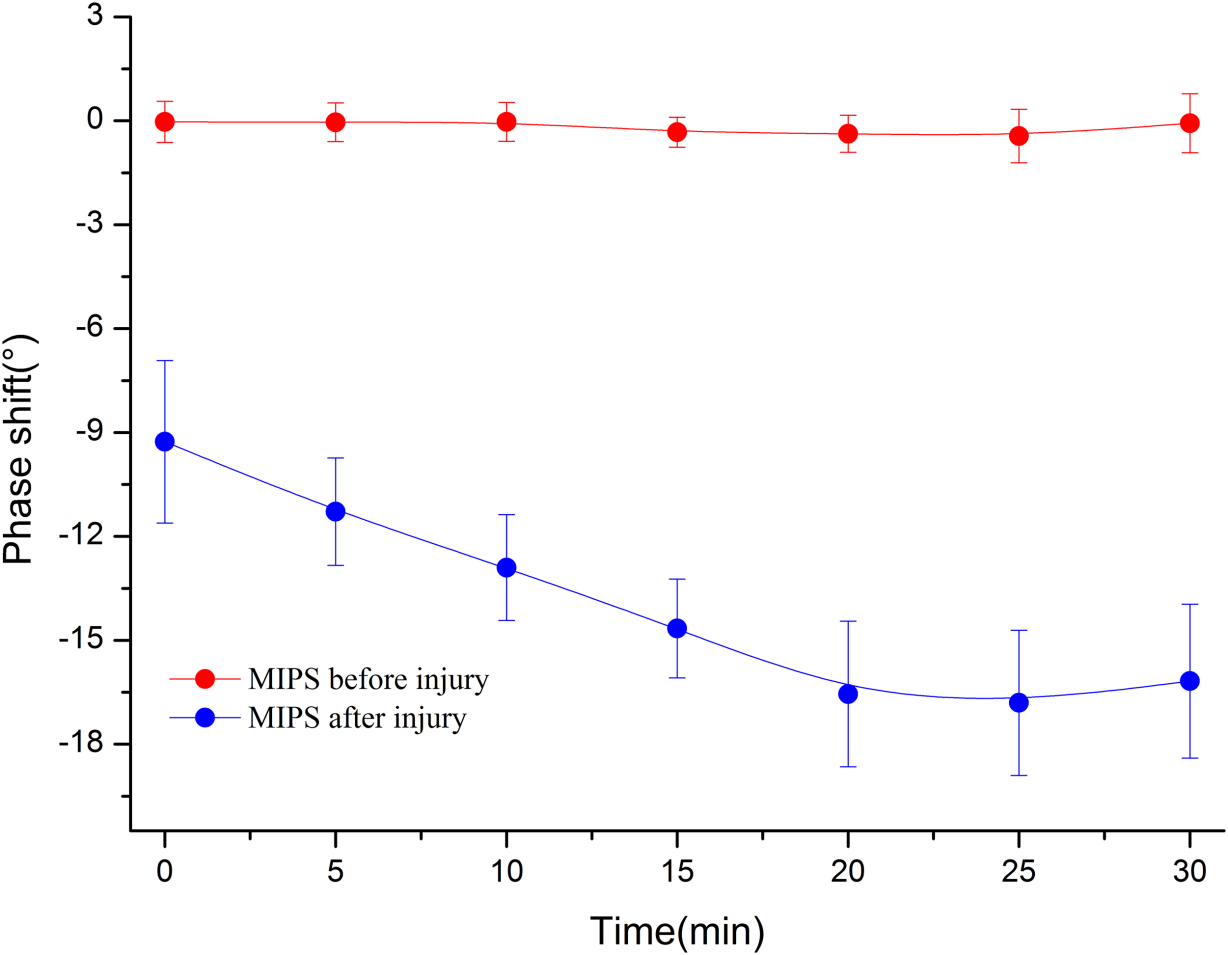
In group 2, the mean ± standard deviation MIPS curve of rabbits declined and then increased slightly after injury, but it remained stable before injury.

Figure 11 presents the mean ± standard deviation trends of the MIPS values at 62.28 ± 1.10 MHz within 30 min before injury and 30 min after injury in group 2. The mean MIPS value was −9.27 ± 2.34° at 0 min and −16.18 ± 2.22° after injury. In contrast, there was no clear change in the MIPS value, which was stable at approximately 0° before injury (mean MIPS value: 0.44 ± 0.77°). Although the trend in the mean MIPS values after injury was similar to that of group 1, it was not completely consistent from 0 to 30 min compared to that of group 1. An inflection point appeared at 25 min (mean MIPS: −16.81 ± 2.10°), then made the mean MIPS values trend upward. A paired *t*-test analysis with *p*-value < 0.05 for the same time before and after injury showed that the MIPS value was significantly different before and after injury. This confirms that the MIPS value can discriminate CCH even if the severity increases (e.g., injured by 650 kPa impact). Additionally, a Pearson correlation coefficient between the mean MIPS value and time was −0.527 before injury (|r| < 0.9). Conversely, it was −0.942 after injury (|r| > 0.9), which reflects CCH expansion over time after injury. Further, the inflection point of the phase curve at 25 min indicates that the mean brain conductivity changes reversed at that time. Hence, the mean brain conductivity decreased before 25 min and then rose after 25 min, which we can infer means that the volume change of certain tissue compositions in the brain reached a critical value, resulting in a reverse change in conductivity at 25 min.

### CCH severity classification

**Figure 12:**
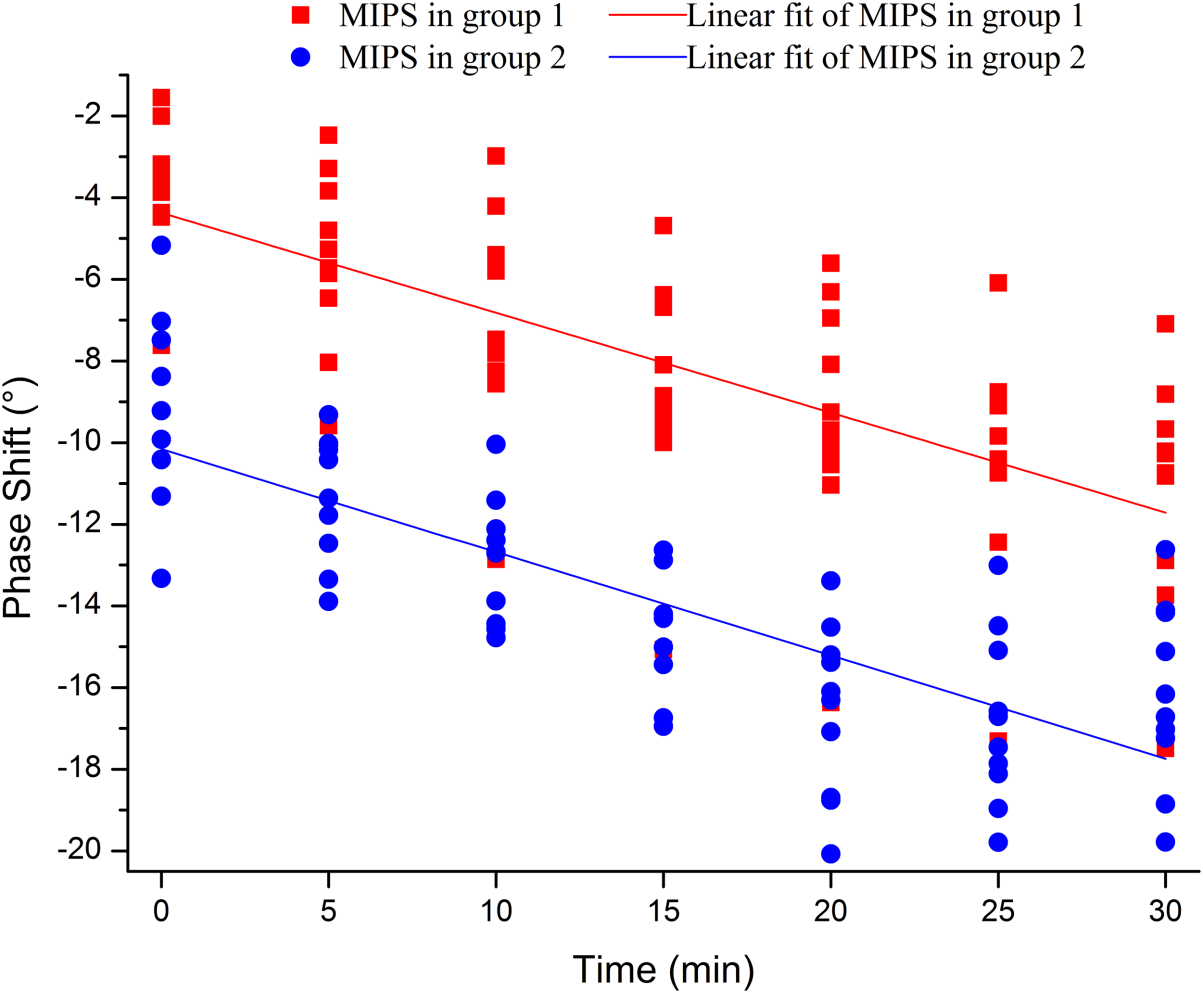
MIPS values of 20 rabbits sampled every 5 min in both two groups within 30 min after injury were plotted as scatter chart. Two linear fits of group 1 and group 2 were calculated by these scatters.

In the study, two CCH severity levels besides the normal condition were established by impacting the head of the rabbit with two different pressure values. First, Fig. 12 shows the scatter chart of the MIPS values plotted in 20 rabbits (Nos. 1–20) within 30 min of the injury. Linear fits of the scatter charts were conducted for both group 1 and group 2. The two linearly fitted lines can be considered to be severity level lines. The paired sample Wilcoxon signed rank test for the MIPS values between group 1 and group 2 shows there was a significant difference in the data for group 1 and group 2 (*p*-value < 0.05). The linear fits of scatter and statistical analysis intuitively and qualitatively confirm that the MIPS value can distinguish the severity of rabbit CCH.

To test the accuracy of CCH severity classification, an SVM classifier-based MATLAB tool (R2015b) was used. There should be two severity levels, one from a 600 kPa impact (group 1) and one from a 650 kPa (group 2) impact. Data came from sampling at seven moments within 30 min in the Nos. 1–20 rabbits (mild group including Nos. 1–10, severe group including Nos. 11–20). At each sampling moment, 20 sets of MIPS data were collected. LOOCV was used for validation in each data set collected at one moment. One data sample was left out as a test sample, the remaining 19 were used as a training set. Each sample of the 20 was picked up in turn. Repeated classifications were performed on each of the 20 data sets that were acquired at one moment, and the accuracy of the MIPS data given in Table 1 is the average result of the 20 repetitions, which shows the classification accuracy of the MIPS data was above 90% for both CCH severity levels.

**Tables 1:** MIPS classification accuracy of two CCH severity levels in rabbits.

Time (min)	0	5	10	15	20	25	30
MIPS accuracy	90%	90%	90%	100%	90%	90%	90%

## Discussion

According to Kim (2011), if TBI patients receive surgical treatment within 4 h of their injury, the mortality rate will decrease significantly. This means time is vital for most CCH patients with a TBI. If patients' brain lesion information can be quickly obtained on the scene after an injury or on the way to the hospital, patients could get scheduled earlier for treatments, which could improve the prognosis of patients. The advantages of the MIPS technique include that it is noncontact, is highly sensitive in the early stage, can evaluate changes in hemorrhage for various locations, can be used as a real-time monitor prior to the hospital, and is expected to perform early clinical diagnoses of TBI-induced CCH.

In this work, CCH is formed by an impact on the selected parietal position of the head in rabbits. After being impacted, mechanical stress and shear forces can cause laceration of the parietal lobe. This can lead to a series of mechanical effects such as extrusion, tearing, and pulling near the brain impact or hedge position, due to the brain movement lagging behind the movement of the skull. Next, the brain tissue collides with the irregular inner surface of the skull to form inertial injuries, and it is also very likely that cavitation effects occur on the base of skull, causing artery rupture (Xiong, Mahmood & Chopp, 2013; Margulies et al., 2015; Oehmichen et al., 2003; Dixon et al., 1994; Davis, 2000). Hence, it is determined that CCH is caused by trauma, which is very different from an intracranial injection of blood that was reported by Li et al. (2017b) and Pan et al. (2015) in their process of forming injuries. The former is a spontaneous hemorrhage, the latter is a passive hemorrhage with a volume of three ml, which may be too large for the cranial cavity of a rabbit (Sawyer, Everett & Green, 1954). The animal model used in the experiment can cause CCH by adjusting the appropriate barometric pressure value on the impactor to control the volume of hemorrhage indirectly, which was verified as feasible in our CT experiment. We can conclude that this animal model has two advantages: one is optional injury location, and another is that it can avoid secondary injury compared to the weight drop model, due to the rabbit being fixed on the universal slab (Xiong, Mahmood & Chopp, 2013). The two advantages can make the injury more repeatable. However, the impactor lacks a sensor that can observe the instantaneous speed and acceleration at the impact location. These sensors are more direct indexes to describe the degree of injury in an animal (Liu et al., 2016; Wu et al., 2016) than the impact pressure, and we will add the sensor in future experiments.

Measurement of MIPS method is sensitive to movement of the target inside the coil Jin et al. (2014a), so a marker was made on the experiment platform by a caliper for rough positioning, and two point sources that emit perpendicular light beam to the side and top of rabbit head for maintaining relatively consistent with coil sensor position when putting the rabbit back again after injury. Further, a verification experiment involved five healthy rabbits that measured the MIPS value of them when their heads inserted into the coil sensor twice. The result showed the difference of the mean MIPS value of the two positionings was approximately 0.37 ± 0.92°, which was much less than the minimal MIPS value of −3.77 ± 1.64° after injury described in the previous section, indicating the change of MIPS value in reentering to the coil sensor can be ignored compared with the MIPS value caused after injury, which did not affect the diagnosis of CCH rabbit in this study.

From the location of hemorrhage accumulation reflected by CT images, it is possible to come from a rupture of the pia mater, arachnoid and brain parenchyma after the impact. A partial hemorrhage on the surface of the brain enters the lateral ventricle through the sagittal sinus, forming an early intracranial hemorrhage with flowing out of the vessel from the brain parenchyma. Since the mean brain conductivity reflected by the MIPS value is closely related to the volume proportion of the intracranial tissue and is positive correlation (Griffiths, Stewart & Gough, 1999), an alteration in the proportion of intracranial tissue volume caused by the continuous accumulation of intracranial hemorrhage can lead to change of conductivity indirectly, which is a pathological process involved in cerebrospinal fluid (CSF) compensation and cerebral blood flow (CBF) compensation (Chen et al., 2017). With the onset of compensation, changes in the proportion of intracranial tissue volume cause changes to the mean brain conductivity. The conductivity values of CSF, blood, gray matter, and white matter are 2.070, 1.210, 0.513, and 0.293 S/m at 65 MHz, respectively (Gabriel, 1996). During the compensation period with the volume of CSF and CBF decreasing, the mean brain conductivity declines and can lead to the MIPS value decreasing, which is consistent with the results of Fig. 9 that describe the MIPS values throughout the measurement period after injury and the result of Fig. 11 describes the MIPS values within 25 min after injury. When the CSF and CBF compensation are exhausted the hemorrhage accumulates continuously to cause a rise in the mean brain conductivity, resulting also in a rise of the MIPS value, which is consistent with the result of Fig. 11 that describes the last 5 min of measurement. Therefore, we can easily find that group 1 did not enter the compensation period, but group 2 entered the compensation period at 25 min, inferring that the CCH injury in group 2 was more severe than it was in group 1.

**Figure 13:**
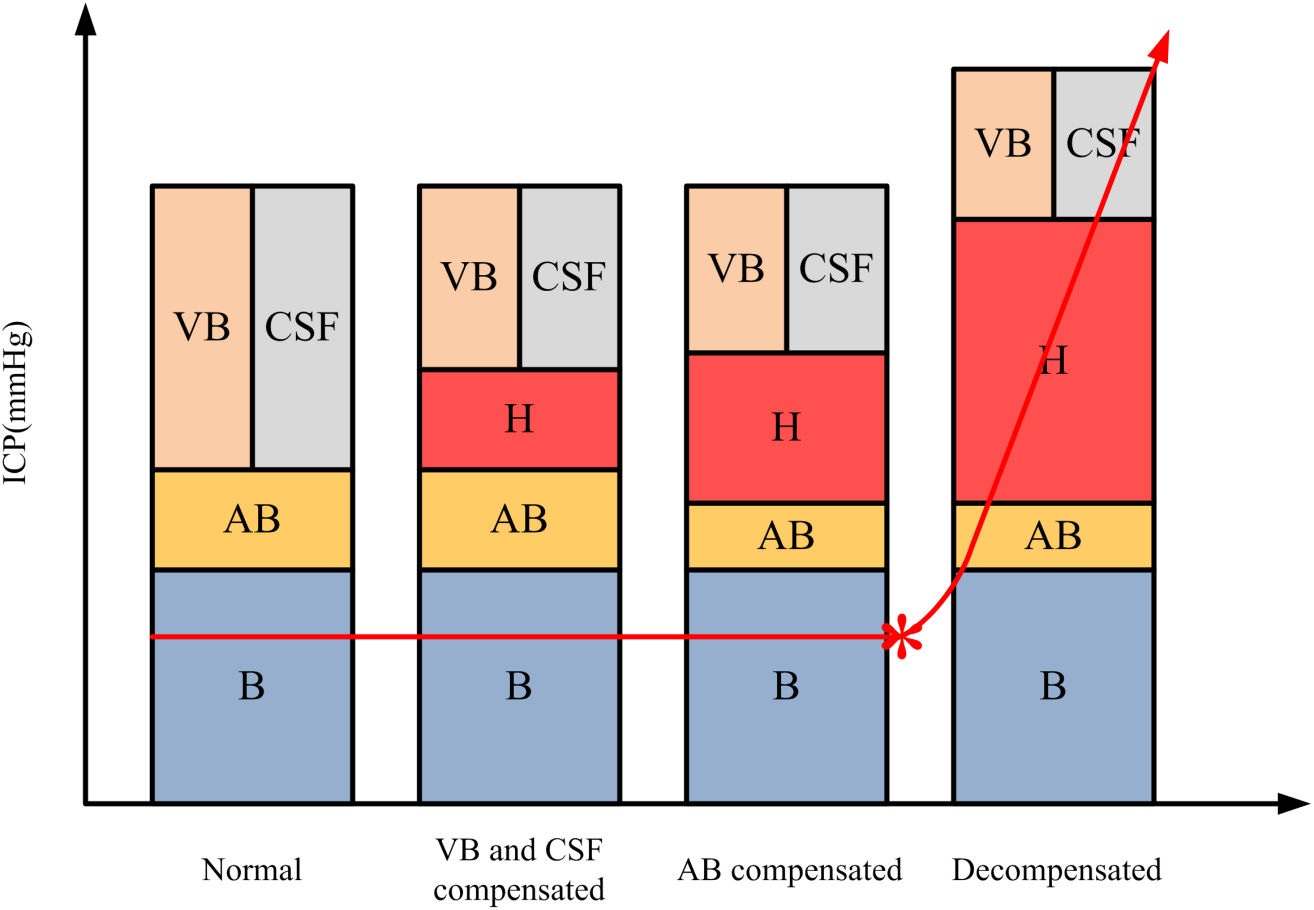
The main tissue content in the skull and ICP curve under normal, compensated, and decompensated conditions (Wykes & Vindlacheruvu, 2015). B, brain; AB, arterial blood; VB, venous blood; H, hemorrhage; CSF, cerebrospinal fluid.

The proportion of intracranial tissue volume changes during cerebral autoregulation can be reflected by a MIPS curve. This demonstrates that the MIPS value is a functional diagnostic technique that can intervene before the appearance of structural brain lesions. Although ICP monitoring is the standard method of detection in the latest TBI guidelines (Carney et al., 2017), and Li et al. (2017a) and Sun et al. (2016) also proved that ICP monitoring has high sensitivity and accuracy in diagnosing brain lesions. Figure 13 from the study of Wykes & Vindlacheruvu (2015) revealed that ICP begins increasing after cerebral autoregulation is exhausted when the brain has entered a critical period. In addition, CT is not only a golden standard for diagnosing intracranial hemorrhage but also a necessary procedure in the trauma center after TBI (Tonui et al., 2018). Nevertheless, CT is limited to detect only structural lesions, but not functional lesions, because of its detection principle, which is based on the difference in the absorption of X-rays by objects so that it can describe abnormal structure anatomy and abnormal contrast enhancement (Hernandez-Maraver et al., 2006). Sherer et al. (2009) reported that the use of volumetric measurements of changes in tissue composition and cortical contusions makes it difficult to achieve the diagnostic accuracy needed for TBI, indicating that CT has some limitations in diagnosing brain functional lesions and may need to be combined with other diagnostic methods to improve its accuracy. As a result, the premise of TBI diagnosis based on CT and ICP monitoring depends on the appearance of structural lesions. However, the MIPS value has proven that it can make a diagnosis in the early stage, which would help to determine if there are functional lesions after TBI, indicating it is a potential, early diagnosis technique for most CCH in TBI.

There are several limitations in this work. First, it was not easy to achieve a quantitative measurement using a CT that serves people instead of animals because intracranial hemorrhages were diffuse in rabbits after their injuries. However, the severity of injury in group 1 and group 2 allowed the location, area and slices of the hemorrhage to be identified, so it can be used as a semiquantitative verification method, which has already achieved its purpose. Second, there is a series of complicated physiopathology pathways in the brain after TBI (Prins et al., 2013; Kaur & Sharma, 2018). During the acute stage after injury (≤1 h), a massive release of glutamate from presynaptic terminals disrupts ionic equilibrium on postsynaptic membranes, leading to an increase in released potassium (K^+^) and calcium (Ca^2+^) accumulation (Ankarcrona et al., 1995; Mark et al., 2001). Analogous processes like those aforementioncal ones may induce a change of the mean brain conductivity, which inturns affect the measurement results (Aronowski & Zhao, 2011; Laufer, Solomon & Rubinsky, 2012). Third, the number of samples is a defect for the stability of the classifier in this experiment, and we will expand the sample size in further work.

## Conclusions

In this study, we made rabbits form two CCH severity levels through an impactor verified by CT, which was a way caused TBI closer to reality. A new diagnostic method for this TBI-induced CCH based on MIPS technology has the advantages of noncontact, portability and continuous monitoring. The MIPS results of CCH revealed that the MIPS value decreases gradually after injury if cerebral autoregulation was not exhausted or if it rises, which is consistent with changes in the mean brain conductivity. Also, linear fitting of the MIPS value scatters more intuitively, which demonstrates the ability of MIPS method to distinguish CCH severity levels. These findings validate that the MIPS method is able to diagnose CCH after TBI, which is a potential technique for prehospital diagnosis. Further investigations include optimizing the coil sensor, which facilitates its capacity in multifrequency measurement.

## Supplemental Information

10.7717/peerj.6717/supp-1 Supplemental Information 1 Average MIPS data per 5 min in 20 rabbits. Raw data exported from the detection system applied for data analyses and preparation for Figs. 8–11 for the time period of 30 min before and after injury.

10.7717/peerj.6717/supp-2 Supplemental Information 2 CT images of group 1 (impact by 600 kPa), group 2 (impact by 650 kPa) and control group. Raw data exported from CT applied for analyses of cerebral hemorrhage in rabbits and preparation for Figs. 5–7.

## Additional Information and Declarations

### Data Availability

The following information was supplied regarding data availability: The raw data are available in the Supplemental Files.
